# Direct oral anticoagulant use and risk of severe COVID‐19

**DOI:** 10.1111/joim.13205

**Published:** 2020-12-19

**Authors:** B. Flam, V. Wintzell, J. F. Ludvigsson, J. Mårtensson, B. Pasternak

**Affiliations:** ^1^ From the Perioperative Medicine and Intensive Care Karolinska University Hospital Stockholm Sweden; ^2^ Section of Anaesthesiology and Intensive Care Medicine Department of Physiology and Pharmacology Karolinska Institutet Stockholm Sweden; ^3^ Clinical Epidemiology Division Department of Medicine Solna Karolinska Institutet Stockholm Sweden; ^4^ Department of Medical Epidemiology and Biostatistics Karolinska Institutet Stockholm Sweden; ^5^ Department of Paediatrics Örebro University Hospital Örebro Sweden; ^6^ Division of Epidemiology and Public Health School of Medicine University of Nottingham Nottingham UK; ^7^ Department of Medicine Columbia University College of Physicians and Surgeons New York NY USA; ^8^ Department of Epidemiology Research Statens Serum Institut Copenhagen Denmark

**Keywords:** anticoagulants, atrial fibrillation, COVID‐19, direct‐acting oral anticoagulants, SARS‐CoV‐2

## Abstract

**Background:**

Hypercoagulability and thromboembolism are prominent features of severe COVID‐19, and ongoing anticoagulant use might be protective.

**Methods:**

We conducted a nationwide register‐based cohort study in Sweden, February through May, 2020, to assess whether ongoing direct oral anticoagulant (DOAC) use was associated with reduced risk of hospital admission for laboratory‐confirmed COVID‐19, or a composite of intensive care unit (ICU) admission or death due to laboratory‐confirmed COVID‐19.

**Results:**

DOAC use (*n *= 103 703) was not associated with reduced risk of hospital admission for COVID‐19 (adjusted hazard ratio [aHR] [95% confidence interval] 1.00 [0.75–1.33] vs. nonuse atrial fibrillation comparator [*n *= 36 875]; and aHR 0.94 [0.80–1.10] vs. nonuse cardiovascular disease comparator [*n *= 355 699]), or ICU admission or death due to COVID‐19 (aHRs 0.76 [0.51–1.12], and 0.90 [0.71–1.15], respectively).

**Conclusion:**

Ongoing DOAC use was not associated with reduced risk of severe COVID‐19, indicating that prognosis would not be modified by early outpatient DOAC initiation.

## Graphical abstract

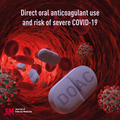

## Introduction

Coronavirus disease 2019 (COVID‐19), caused by severe acute respiratory syndrome coronavirus 2 (SARS‐CoV‐2), is associated with hypercoagulability. Thrombosis is common in patients hospitalized with COVID‐19 [1] and seems to play a key role both in the pathophysiology [2], and as incident complications [3].

Given the thrombogenic features of COVID‐19 potentially predisposing for a progressive disease course, it is plausible that anticoagulation may protect against severe disease. Currently, pharmacological parenteral thromboprophylaxis is widely advocated for patients hospitalized for COVID‐19 [4, 5], although the efficacy of this intervention on further disease progression and prognosis is unknown and the subject of ongoing trials. Whether long‐term anticoagulation with therapeutic doses initiated before SARS‐CoV‐2 infection influences COVID‐19 progression is unclear. To date, eight mostly small regional or single‐centre studies of a total of 434 patients with preexisting anticoagulant use have been reported, presenting inconclusive and inconsistent results [6, 7, 8, 9, 10, 11, 12, 13].

We conducted a nationwide register‐based cohort study in Sweden to investigate whether ongoing direct oral anticoagulant (DOAC) use was associated with reduced risk of severe COVID‐19.

## Materials and methods

This cohort study was based on nationwide Swedish registers. Patients with DOAC use and nonvalvular atrial fibrillation or flutter (AF) were compared with two nonuse comparator groups: patients with AF, and patients with major cardiovascular disease (CVD). We assessed the risk of two co‐primary outcomes reflecting severe forms of COVID‐19: hospital admission for COVID‐19, and the composite of intensive care unit (ICU) admission or death due to COVID‐19. The Swedish Ethical Review Authority approved the study (number 2020‐02536).

### Data sources

Data sources included the National Patient Register, which accumulates data on all hospital admissions and outpatient and emergency department visits in Sweden, including physician‐assigned diagnoses coded according to the International Classification of Diseases, 10^th^ Revision, Swedish Edition (ICD‐10‐SE) and procedure codes (positive predictive value of AF diagnosis record, 97%) [14]; the Swedish Prescribed Drug Register, which captures detailed information on all prescriptions filled at all Swedish pharmacies; Statistics Sweden’s sociodemographic data; the Cause of Death Register, which captures dates and causes of death based on death certificates; and the Swedish Intensive Care Registry, which is a national registry that captures close to all adult ICU admissions in the country.

### Study cohort

We constructed a cohort consisting of all individuals in Sweden aged 45 to 84 years and alive through 31 January 2020 (which was the date of the first confirmed COVID‐19 case in the country), with a recorded diagnosis of AF, ischaemic heart disease, heart failure or cardiomyopathy, stroke or transient ischaemic attack, systemic thromboembolism, or vascular disease (Table S1), who had resided in Sweden throughout the last year and had at least one drug prescription or specialist care contact within the previous three years (to ensure healthcare system activity). To minimize bias, including exposure misclassification, patients were excluded in case of DOAC use with non‐AF indication; recent but ceased DOAC use; possible DOAC contraindication; recent warfarin use or likely warfarin indication, including mechanical heart valve or mitral stenosis; and severe illness. Further exclusion criteria were recent hospital admission; recent venous thromboembolism; and recent use of heparins. Exclusion criteria are specified in Table S2.

### DOAC use and comparator groups

Ongoing use of a DOAC (dabigatran, apixaban, rivaroxaban or edoxaban) was defined as a prescription for any of these drugs that was filled before the index date of 1 February 2020 and whose duration overlapped the index date (Table S3). The estimated duration of filled prescriptions was defined based on the number of tablets dispensed, allowing for a gap of 30 days. Nonuse was defined as no DOAC use within 12 months before the index date. Once defined as a DOAC user or nonuser on the index date, a patient was considered to belong to that group throughout follow‐up.

The exposed group consisted of patients with nonvalvular AF with ongoing DOAC use. An active comparator design was unfeasible in this setting. In the interest of robustness and consistency of study findings, two different nonuse comparator groups were used: patients with AF who were nonusers of DOAC, and patients with CVD (AF, ischaemic heart disease, heart failure/cardiomyopathy, stroke, transient ischaemic attack, systemic thromboembolism, or vascular disease) who were nonusers of DOAC. By design, the nonuse AF comparator group was thus a subgroup of the nonuse CVD comparator group.

### Outcomes

The two co‐primary outcomes were hospital admission for laboratory‐confirmed COVID‐19, defined as the first hospital admission with primary diagnosis ICD‐10‐SE code U07.1 (COVID‐19, virus identified), and the composite of ICU admission or death due to laboratory‐confirmed COVID‐19, which was defined as an ICU admission during a hospitalization with a primary diagnosis U07.1, or death where U07.1 was recorded as the underlying cause or death of any cause within 30 days of a hospital admission with a primary diagnosis U07.1.

Prespecified secondary analyses assessed both co‐primary outcomes according to DOAC subtype (direct thrombin inhibitor [dabigatran], or direct factor Xa inhibitor [apixaban, rivaroxaban or edoxaban]), and the individual components of the second co‐primary outcome (ICU admission and death due to COVID‐19, respectively). In a sensitivity analysis, we assessed the risk of all‐cause mortality to examine the potential for residual confounding.

### Statistical analysis

We used Cox proportional‐hazards regression, estimating the following models: unadjusted; adjusted for age and sex; and fully adjusted. The fully adjusted multivariable models included 42 potential confounders including age, sex, sociodemographic factors, comorbidities, medications, and healthcare utilization (Table S4). Hazard ratios (HRs) were considered statistically significant if the 95% confidence intervals (CIs) did not contain 1.0. Adjusted absolute risk differences were calculated as [adjusted HR (aHR)–1]×crude risk amongst the unexposed. In the analysis of hospital admission for COVID‐19, patients were followed from cohort entry until outcome event, end of study period or any‐cause death, whichever occurred first. In the analysis of ICU admission or death due to COVID‐19, patients were followed from cohort entry until outcome event, end of study period or other‐cause death, whichever occurred first. All analyses were performed using SAS, version 9.4 (SAS Institute Inc.).

## Results

### Cohort and exposure/comparator groups

**Fig. 1:**
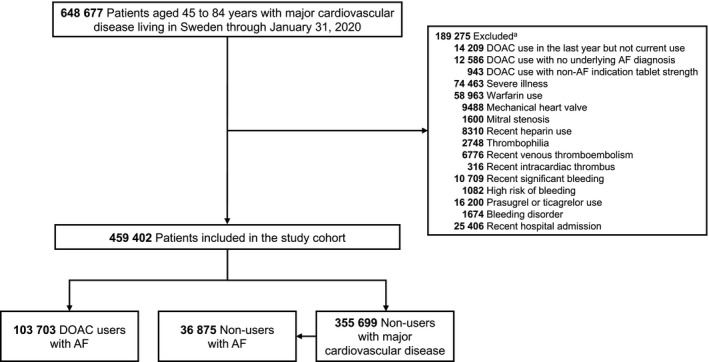
Cohort construction flow chart. AF, atrial fibrillation; DOAC, direct oral anticoagulant. ^a^ Numbers of excluded patients do not accumulate to the total sum, since some patients were excluded for more than one reason.

The study cohort eligibility criteria were met in 459 402 patients (Fig. 1). Of these, 103 703 were patients with nonvalvular AF who were DOAC users (93 354 [90.0%] using direct factor Xa inhibitors). The comparator groups included 36 875 patients with AF with no DOAC use and 355 699 patients with CVD with no DOAC use.

Baseline patient characteristics are shown in Table 1. Compared with AF patients with no DOAC use, DOAC users were older and more often female, had more somatic comorbidities, specialist care consumption, and medications except for antiplatelet agents and nonsteroidal anti‐inflammatory drugs. When compared with the CVD group with no DOAC use, DOAC users were older, but had a lower prevalence of ischaemic heart disease and cerebrovascular disease/systemic thromboembolism, whilst having higher specialist care consumption and medication use except for antiplatelet agents, statins, and nonsteroidal anti‐inflammatory drugs. Overall, the median follow‐up time during the four‐month study period was 121.0 days (interquartile range, 121.0–121.0 days).

**Table 1:** Baseline patient characteristics^a^

Characteristic	DOAC use, AF (*n *= 103 703)	Comparator groups	
		No DOAC use, AF (*n *= 36 875)	No DOAC use, major CVD (*n *= 355 699)
Male sex, *n *(%)	62 488 (60.3)	25 020 (67.9)	214 041 (60.2)
Age, mean (SD), years	73.6 (7.6)	66.4 (10.5)	69.3 (9.6)
Age group,* n *(%)			
45–49 years	739 (0.7)	2648 (7.2)	12 872 (3.6)
50–54 years	1918 (1.8)	3785 (10.3)	22 531 (6.3)
55–59 years	3747 (3.6)	4723 (12.8)	32 991 (9.3)
60–64 years	7500 (7.2)	5832 (15.8)	44 929 (12.6)
65–69 years	14 974 (14.4)	4987 (13.5)	56 036 (15.8)
70–74 years	24 900 (24.0)	5677 (15.4)	72 449 (20.4)
75–79 years	27 557 (26.6)	5149 (14.0)	66 033 (18.6)
80–84 years	22 368 (21.6)	4074 (11.0)	47 858 (13.5)
DOAC, *n *(%)			
Dabigatran	10 349 (10.0)	NA	NA
Apixaban	72 347 (69.8)	NA	NA
Rivaroxaban	18 781 (18.1)	NA	NA
Edoxaban	2226 (2.1)	NA	NA
Time since first DOAC dispensing, *n *(%)			
0–6 months	7394 (7.1)	NA	NA
7–24 months	29 012 (28.0)	NA	NA
>24 months	67 245 (64.8)	NA	NA
Place of birth, *n *(%)			
Scandinavia	96 685 (93.2)	33 410 (90.6)	313 295 (88.1)
Rest of Europe	4922 (4.7)	2008 (5.4)	21 939 (6.2)
Outside Europe	2095 (2.0)	1455 (3.9)	20 448 (5.7)
Civil status, *n *(%)			
Married/living with partner	57 437 (55.4)	19 593 (53.1)	185 901 (52.3)
Single	46 266 (44.6)	17 282 (46.9)	169 798 (47.7)
Education, *n *(%)			
Primary/secondary school, vocational training	73 138 (70.5)	24 208 (65.6)	259 527 (73.0)
Short tertiary education	12 582 (12.1)	5035 (13.7)	41 077 (11.5)
Medium or long tertiary education	17 047 (16.4)	7333 (19.9)	51 774 (14.6)
Comorbidities, *n *(%)			
Ischaemic heart disease	24 519 (23.6)	7320 (19.9)	167 019 (47.0)
Heart failure/cardiomyopathy	26 544 (25.6)	4442 (12.0)	46 889 (13.2)
Valve disorder	11 456 (11.0)	3611 (9.8)	20 249 (5.7)
Ischaemic stroke/TIA/systemic thromboembolism	17 650 (17.0)	2853 (7.7)	102 916 (28.9)
Haemorrhagic/unspecified stroke	6451 (6.2)	1849 (5.0)	35 789 (10.1)
Other vascular disease	8112 (7.8)	2540 (6.9)	59 007 (16.6)
Arrhythmia (other than AF/flutter)	18 264 (17.6)	7357 (20.0)	27 735 (7.8)
Lung disease	21 762 (21.0)	6093 (16.5)	61 856 (17.4)
Renal disease	6082 (5.9)	1741 (4.7)	14 354 (4.0)
Liver disease	1445 (1.4)	832 (2.3)	6613 (1.9)
Venous thromboembolism (>1 year prior)	5692 (5.5)	1390 (3.8)	12 196 (3.4)
Malignancy (>1 year prior)	10 826 (10.4)	3210 (8.7)	30 589 (8.6)
Peptic ulcer disease (>90 days prior)	2550 (2.5)	1001 (2.7)	9316 (2.6)
Psychiatric disorder/substance abuse	14 249 (13.7)	6994 (19.0)	68 861 (19.4)
Prescription drug use, *n *(%)			
Aspirin	3514 (3.4)	9211 (25.0)	198 617 (55.8)
P2Y_12_ inhibitor (excl. prasugrel/ticagrelor)	1576 (1.5)	1104 (3.0)	48 929 (13.8)
ACE inhibitor/ARB	65 066 (62.7)	13 357 (36.2)	203 516 (57.2)
Calcium‐channel blocker	28 913 (27.9)	6571 (17.8)	103 639 (29.1)
Loop diuretic	21 484 (20.7)	2961 (8.0)	29 834 (8.4)
Other diuretic	19 880 (19.2)	2972 (8.1)	41 863 (11.8)
Beta‐blocker	83 454 (80.5)	19 020 (51.6)	170 370 (47.9)
Statin	48 104 (46.4)	10 297 (27.9)	219 559 (61.7)
Metformin	13 974 (13.5)	2848 (7.7)	50 626 (14.2)
Insulin	6687 (6.4)	1552 (4.2)	26 906 (7.6)
Other glucose‐lowering drug	8634 (8.3)	1678 (4.6)	30 435 (8.6)
Antidepressant/antipsychotic	14 888 (14.4)	5205 (14.1)	60 606 (17.0)
Beta_2_‐agonist inhalant	7828 (7.5)	2099 (5.7)	24 769 (7.0)
Anticholinergic inhalant	6354 (6.1)	1444 (3.9)	18 944 (5.3)
Glucocorticoid inhalant	9827 (9.5)	2647 (7.2)	28 930 (8.1)
Oral glucocorticoid	8959 (8.6)	2071 (5.6)	22 792 (6.4)
NSAID	3563 (3.4)	3277 (8.9)	29 036 (8.2)
Opioid	12 137 (11.7)	3458 (9.4)	38 406 (10.8)
Healthcare utilization in the last year, *n *(%)			
Specialist care outpatient visits			
0	23 507 (22.7)	13 728 (37.2)	128 877 (36.2)
1–3	47 814 (46.1)	16 038 (43.5)	153 267 (43.1)
>3	32 382 (31.2)	7109 (19.3)	73 555 (20.7)
Hospital admissions			
0	75 301 (72.6)	31 192 (84.6)	293 656 (82.6)
1	17 366 (16.7)	3581 (9.7)	41 528 (11.7)
>1	11 036 (10.6)	2102 (5.7)	20 515 (5.8)
Prescription drugs			
0–5	21 999 (21.2)	19 121 (51.9)	112 239 (31.6)
6–10	42 370 (40.9)	10 371 (28.1)	137 551 (38.7)
11–15	24 638 (23.8)	4771 (12.9)	68 478 (19.3)
>15	14 696 (14.2)	2612 (7.1)	37 431 (10.5)

Percentages may not total 100 because of rounding.

ACE, angiotensin‐converting enzyme; AF, atrial fibrillation; ARB, angiotensin‐receptor blocker; CVD, cardiovascular disease; DOAC, direct oral anticoagulant; NA, not applicable; NSAID, nonsteroidal antiinflammatory drug; SD, standard deviation; TIA, transient ischaemic attack.

^a^ Geographic baseline patient characteristics are shown in Table S5.

### Risk of severe COVID‐19

Table 2 shows results of analyses of the co‐primary outcomes. There were 360 hospital admissions for COVID‐19 amongst the DOAC users (crude risk, 0.35%), vs. 95 amongst nonusers with AF (0.26%) and 1119 amongst nonusers with CVD (0.31%). In the fully adjusted multivariable analysis, DOAC use, as compared with nonuse, was not associated with reduced risk of hospital admission for COVID‐19 (aHR, 1.00; 95% CI, 0.75–1.33 vs. nonuse AF comparator, and 0.94; 95% CI, 0.80–1.10 vs. nonuse CVD comparator).

**Table 2:** Risk of severe COVID‐19 amongst DOAC users vs. nonuse comparators

Outcome	Patients, *n*	Events, *n* (%)	Unadjusted hazard ratio (95% CI)	Hazard ratio (95% CI) adjusted for age and sex	Fully adjusted^a^ hazard ratio (95% CI)	Fully adjusted^a^ absolute risk difference, % (95% CI)
Hospital admission for COVID‐19						
DOAC use	103 703	360 (0.35)	—	—	—	—
vs. nonuse AF comparator	36 875	95 (0.26)	1.35 (1.07–1.69)	1.14 (0.89–1.45)	1.00 (0.75–1.33)	0.00 (–0.07–0.09)
vs. nonuse CVD comparator	355 699	1119 (0.31)	1.10 (0.98–1.24)	1.04 (0.92–1.17)	0.94 (0.80–1.10)	–0.02 (–0.06–0.03)
ICU admission or death due to COVID‐19						
DOAC use	103 703	161 (0.16)	—	—	—	—
vs. nonuse AF comparator	36 875	55 (0.15)	1.04 (0.77–1.41)	0.71 (0.52–0.98)	0.76 (0.51–1.12)	–0.04 (–0.07–0.02)
vs. nonuse CVD comparator	355 699	473 (0.13)	1.17 (0.98–1.40)	0.97 (0.81–1.17)	0.90 (0.71–1.15)	–0.01 (–0.04–0.02)

AF, atrial fibrillation; CI, confidence interval; COVID‐19, coronavirus disease 2019; CVD, cardiovascular disease; DOAC, direct oral anticoagulant; ICU, intensive care unit.

^a^ Adjusted for 42 potential confounders, including age, sex, sociodemographic factors, comorbidities, medications and healthcare utilization (Table S4).

One hundred sixty‐one composite ICU admission or death due to COVID‐19 outcome events occurred amongst the DOAC users (crude risk, 0.16%), vs. 55 amongst nonusers with AF (0.15%) and 473 amongst nonusers with CVD (0.13%). In the fully adjusted multivariable analysis, DOAC use was not associated with a reduced risk of ICU admission or death due to COVID‐19 (aHR, 0.76; 95% CI, 0.51–1.12 vs. nonuse AF comparator, and 0.90; 95% CI, 0.71–1.15 vs. nonuse CVD comparator).

### Additional analyses

Estimates for both co‐primary outcomes were similar for both DOAC subtypes (direct thrombin inhibitor and direct factor Xa inhibitors) (Table S6). Analyses of the individual components of the second co‐primary outcome yielded aHRs of 1.06; 95% CI, 0.48–2.35 vs. nonuse AF comparator, and 0.86; 95% CI, 0.55–1.34 vs. nonuse CVD comparator for ICU admission due to COVID‐19; and 0.72; 95% CI, 0.47–1.10 vs. nonuse AF comparator, and 0.91; 95% CI, 0.70–1.17 vs. nonuse CVD comparator for death due to COVID‐19 (Table S7). In the sensitivity analysis of all‐cause mortality, the aHRs were 0.62; 95% CI, 0.53–0.73 vs. nonuse AF comparator, and 0.79; 95% CI, 0.71–0.87 vs. nonuse CVD comparator (Table S8).

## Discussion

In this nationwide register‐based cohort study including more than 100 000 DOAC users, ongoing use of this class of anticoagulants was not associated with a reduced risk of the two co‐primary outcomes hospital admission for COVID‐19 and a composite of ICU admission or death due to COVID‐19. These findings were consistent in analyses with two different comparator groups, as well as across DOAC subtypes.

In the light of the current pandemic, measures to prevent related morbidity and mortality are much looked‐for. Identified as a key feature of severe COVID‐19, large focus has been put on managing hypercoagulability in hospitalized patients, with interim guidelines supportive of anticoagulation [4, 5]. Preliminary retrospective data ratify these recommendations [15], although results may be biased [16]. Seemingly, the thrombotic disease processes could have commenced already prior to hospital admission, as studies have found early event occurrence (at or within 24 hours of admission) in approximately half of COVID‐19 cases with associated venous thromboembolism [17, 18]. Pulmonary and extra‐pulmonary microvascular thrombosis may considerably contribute to the acute lung injury and multiple organ dysfunction that leads to disease progression and ensuing hospitalization, critical illness, and death. Preemptive anticoagulant treatment before or at the time of SARS‐CoV‐2 infection to protect against severe disease is theoretically appealing but real‐world data have been lacking. Previously, only small studies of COVID‐19 patient cohorts have been conducted showing mixed results [6, 7, 8, 9, 10, 11, 12, 13]. An additional case–control study aimed at investigating the association between renin–angiotensin–aldosterone system blockers and COVID‐19 reported also on oral anticoagulant use (odds ratio, 1.16; 95% CI 1.04–1.30) [19]. The results from the present large observational study do not support that DOAC administration reduces the risk of severe COVID‐19. Rather than against secondary hypercoagulability, therapies may be better directed against thrombogenic inflammation or vasculopathy, but further investigation is required.

This study has strengths and limitations. The nationwide register‐based design enabled the inclusion of over 100 000 DOAC‐exposed individuals and complete follow‐up. The analyses controlled for a large number of potential confounders. Still, pharmacoepidemiologic study designs that utilize a nonuser comparator may be sensitive to confounding. This is however less likely. First, the results were consistent across analyses with two disparate comparator groups exhibiting different characteristics, why confounding would have had to act similarly in both analyses. Second, for confounding to lead to the observation of false‐neutral results in a scenario where a true protective association exists, confounding would have to skew HRs upwards. Thus, it would have to be an unmeasured factor that was more common amongst DOAC users and that led to increased risk of severe COVID‐19 outcomes. Such a factor could be frailty and would be expected to also lead to increased risk of all‐cause mortality. This was not the case; in a sensitivity analysis, DOAC use was associated with a reduced risk of all‐cause mortality. The estimated aHRs for all‐cause mortality are well in line with those from previous real‐world studies comparing DOACs with vitamin K antagonists [20]. Last, thromboprophylaxis administered during hospitalization could not be assessed and may have introduced bias towards the null. This is less likely since it cannot have affected the outcome of hospital admission for COVID‐19.

## Conclusion

In this large nationwide cohort study, there was no significant association between ongoing DOAC use and risk of severe COVID‐19. In search of therapeutics, these findings indicate that COVID‐19 prognosis would not be modified by early outpatient DOAC initiation.

## Author contribution

**Benjamin Flam:** Conceptualization (equal); Investigation (equal); Methodology (equal); Project administration (equal); Writing‐original draft (lead); Writing‐review & editing (equal). **Viktor Wintzell:** Conceptualization (equal); Data curation (lead); Formal analysis (lead); Investigation (equal); Methodology (equal); Writing‐review & editing (equal). **Jonas Ludvigsson:** Conceptualization (equal); Investigation (equal); Methodology (equal); Writing‐original draft (supporting); Writing‐review & editing (equal). **Johan Mårtensson:** Conceptualization (equal); Funding acquisition (equal); Investigation (equal); Methodology (equal); Writing‐review & editing (equal). **Björn Pasternak:** Conceptualization (equal); Funding acquisition (equal); Investigation (equal); Methodology (equal); Project administration (equal); Supervision (lead); Writing‐original draft (supporting); Writing‐review & editing (equal).

## Conflicts of interest

Dr. Ludvigsson coordinates a study on behalf of the Swedish Inflammatory Bowel Disease Register (SWIBREG). This study has received funding from Janssen Corporation. The remaining authors declare no competing financial interests.

## Supporting information


**Table S1.** Study cohort inclusion diagnoses.
**Table S2.** Study cohort exclusion criteria.
**Table S3.** Study drugs.
**Table S4.** Covariates included in multivariable analyses.
**Table S5.** Geographic baseline patient characteristics.
**Table S6.** Risk of severe COVID‐19 among DOAC users vs. non‐use comparators according to DOAC subtype.
**Table S7.** Risk of ICU admission and death due to COVID‐19 analyzed separately.
**Table S8.** All‐cause mortality among DOAC users vs. non‐use comparator groups.
