# Supplementary material for: Direct oral anticoagulant use and risk of severe COVID‐19
Source: J Intern Med. 2020 Dec 19;289(3):411–9. doi: 10.1111/joim.13205 (PMC7753564; doi:10.1111/joim.13205)
Supplement: Supplementary file 1 — Table S1. Study cohort inclusion diagnoses. Table S2. Study cohort exclusion criteria. Table S3. Study drugs. Table S4. Covariates included in multivariable analyses. Table S5. Geographic baseline patient characteristics. Table S6. Risk of severe COVID‐19 among DOAC users vs. non‐use comparators according to DOAC subtype. Table S7. Risk of ICU admission and death due to COVID‐19 analyzed separately. Table S8. All‐cause mortality among DOAC users vs. non‐use comparator groups. [file JOIM-289-411-s001.docx]

# Supplementary Material

**Direct oral anticoagulant use and risk of severe COVID-19**

Benjamin Flam, MD; Viktor Wintzell, MSc; Jonas F. Ludvigsson, MD, PhD; Johan Mårtensson, MD, PhD; Björn Pasternak, MD, PhD

## Table of Contents

[Table S1. Study cohort inclusion diagnoses 2](#_Toc54608422)

[Table S2. Study cohort exclusion criteria 3](#_Toc54608423)

[Table S3. Study drugs 4](#_Toc54608424)

[Table S4. Covariates included in multivariable analyses 5](#_Toc54608425)

[Table S5. Geographic baseline patient characteristics 7](#_Toc54608426)

[Table S6. Risk of severe COVID-19 among DOAC users vs. non-use comparators according to DOAC subtype 8](#_Toc54608427)

[Table S7. Risk of ICU admission and death due to COVID-19 analyzed separately 9](#_Toc54608428)

[Table S8. All-cause mortality among DOAC users vs. non-use comparator groups 10](#_Toc54608429)

**Table S1. Study cohort inclusion diagnoses^a^**

| **Diagnosis category** | **ICD-10-SE code** |
| --- | --- |
| Heart failure/cardiomyopathy | I11.0, I13.x (not I13.1), I42.x, I43.x, I50.x |
| Ischemic stroke/TIA/systemic thromboembolism | G45.x (not G45.4), I63.x, I69.3, I74.x |
| Hemorrhagic/unspecified stroke | I60.x-I62.x, I64.9, I69.0, I69.1, I69.2 |
| Ischemic heart disease | I20.x-I25.x |
| Other vascular disease | E10.5, E11.5, E12.5, E13.5, E14.5, I65.x-I68.x (not I67.6), I69.4, I69.8, I70.x-I77.x (not I74.x), I79.x (not I79.8) |
| Atrial fibrillation or flutter | I48.x |

ICD-10-SE, International Classification of Diseases, 10^th^ Revision, Swedish Edition; TIA, transient ischemic attack

^a^ At least one inpatient or outpatient record (primary or secondary position) of any of the listed diagnoses between February 1, 2010, through January 31, 2020 (10-year look-back), as identified from the National Patient Register, was required for study eligibility assessment.

**Table S2. Study cohort exclusion criteria**

| **Characteristic** | **Time frame (before index date)** | **ICD-10-SE code** | **Procedure code (KVÅ)** | **ATC code** |
| --- | --- | --- | --- | --- |
| Severe illness (ie, dementia, severe chronic kidney disease, severe liver disease, solid organ transplantation, or active malignancy) | 10 years | F00.x-F03.x, F05.1, F10.7A, G30.x, G31.1, G31.8A, I85.x, I86.4, I98.2, K70.4, K71.1, K71.7, K72.1, K72.9, K76.5, K76.6, K76.7, N18.4, N18.5, N25.0, T86.1, T86.2, T86.3, T86.4, Z94.0, Z94.1, Z94.2, Z94.3, Z94.4, Z99.2 | DJ008, DR016, DR024, DR060, DR061; FQx, JAK10, JJCxx, KASxx, TJA33 |  |
|  | 1 year | C00.x-C97.x (not C44.x) |  |  |
| DOAC use in the last year but not ongoing use | 1 year |  |  | B01AE07, B01AF01, B01AF02, B01AF03 |
| DOAC use with no underlying AF diagnosis | 10 years | No I48.x |  | B01AE07, B01AF01, B01AF02, B01AF03 |
| DOAC use with non-AF indication tablet strength | 6 months |  |  | B01AE07 with tablet strength 75 mg, B01AF01 with tablet strength 2.5 mg |
| Warfarin use | 1 year |  |  | B01AA03 |
| Mechanical heart valve | 10 years | Z95.2 | FMD00, FKD00, FGE00, FJF00 |  |
| Mitral stenosis | 10 years | I05.0, I05.2, I34.2, Q23.2 |  |  |
| Recent heparin (incl. low-molecular weight heparin and antithrombin III) use | 90 days |  |  | B01AB |
| Thrombophilia | 10 years | D68.5, D68.6 |  |  |
| Recent venous thromboembolism | 1 year | I26.x, I63.6, I67.6, I80.x-I82.x, O22.3, O22.5, O87.1, O87.3, O88.2 |  |  |
| Recent intracardiac thrombus | 1 year | I23.6, I51.3 |  |  |
| Recent significant bleeding (ie, intracranial [incl. ocular], gastrointestinal, or other major bleeding) | 90 days | A96.x, A98.x, D62.9, D68.3, D69.8, D69.9, H11.3, H35.2, H43.1, I60.x-I62.x, I98.3, J94.2, K22.6, K25.x-K28.x (not K25.3, K25.7, K25.9, K26.3, K26.7, K26.9, K28.3, K28.7, K28.9), K29.0, K62.5, K66.1, K76.2, K92.0, K92.1, K92.2, N02.x, N92.4, N95.0, O03.1, O03.6, O44.1, R04.x, R31.9, R58.9, S06.3, S06.4, S06.5, S06.6, T81.0 |  |  |
| High risk of bleeding (ie, recent thrombocytopenia, recent esophageal varices, or recent peptic ulcer) | 90 days | D69.4, D69.5, D69.6, I98.3, K25.3, K25.7, K25.9, K26.3, K26.7, K26.9, K28.3, K28.7, K28.9 |  |  |
| Prasugrel or ticagrelor use | 6 months |  |  | B01AC22, B01AC24 |
| Bleeding disorder | 10 years | D66.9, D67.9, D68.0, D68.1, D68.2, D68.4, D69.1, D69.3 |  |  |
| Recent hospital admission | 30 days | Admission record in National Inpatient Register |  |  |

AF, atrial fibrillation or flutter; ATC, Anatomical Therapeutic Chemical classification system; DOAC, direct oral anticoagulant; ICD-10-SE, International Classification of Diseases, 10^th^ Revision, Swedish Edition; KVÅ, Classification of Care Measures

**Table S3. Study drugs**

| **Drug** | **Subtype** | **ATC code** |
| --- | --- | --- |
| Dabigatran | Direct thrombin inhibitor | B01AE07 |
| Apixaban | Direct factor Xa inhibitor | B01AF02 |
| Rivaroxaban | Direct factor Xa inhibitor | B01AF01 |
| Edoxaban | Direct factor Xa inhibitor | B01AF03 |

ATC, Anatomical Therapeutic Chemical classification system

**Table S4. Covariates included in multivariable analyses**

| **Variable** |  | |
| --- | --- | --- |
| **Sociodemographic characteristics^a^** |  | |
| Sex |  | |
| Age group (5-year age bands) |  | |
| Health care region of residence (Stockholm, Västra Götaland, Skåne, Östergötland, Jönköping County, Gävleborg, Uppsala, Dalarna, Halland, Sörmland, Värmland, Norrbotten, Västmanland, Örebro County, Kalmar County, Västerbotten, Västernorrland, Blekinge, Kronoberg, Jämtland Härjedalen, Gotland) |  | |
| Population density of municipality (Major city, Commuter municipality close to major city, Large town, Commuter municipality close to large town, Small town, Commuter municipality close to small town, Rural municipality with tourism sector, Rural municipality) |  | |
| Place of birth (Scandinavia, Rest of Europe, Outside Europe)^b^ |  | |
| Civil status (Married/living with partner, Single) |  | |
| Education (Primary/secondary school/vocational training, Short tertiary education, Medium/long tertiary education)^b^ |  | |
| **Comorbidities (10-year look-back)^c^** | **ICD-10-SE code** | **Procedure code (KVÅ)** |
| Ischemic heart disease | I20.x-I25.x, Z95.1, Z95.5 | FNA-FNG; DF009, DF019, DF020 |
| Heart failure/cardiomyopathy | I11.0, I13.x (not I13.1), I42.x, I43.x, I50.x, J81.9 |  |
| Valve disorder | I05.1, I05.8, I05.9, I06.x-I08.x, I34.x-I37.x (not I34.2), Q22.x, Q23.x (not Q23.2) |  |
| Ischemic stroke/TIA/systemic thromboembolism | G45.x (not G45.4), H34.x (not H34.8, H34.9), I63.x, I69.3, I74.x, Z86.6A, Z86.6B |  |
| Hemorrhagic/unspecified stroke | I60.x-I62.x, I64.9, I69.0, I69.1, I69.2, Z86.7C |  |
| Other vascular disease | E10.5, E11.5, E12.5, E13.5, E14.5, I70.x-I77.x (not I74.x), I79.x (not I79.8), K55.0, K55.1 |  |
| Arrhythmia (other than AF/flutter) | I44.1, I44.2, I45.3, I45.6, I46.x, I47.x, I49.x |  |
| Lung disease | D86.0, D86.1, E66.2, E84.0, G47.3, I27.x, J40.x-J45.x, J46.9, J47.x, J60.x-J67.x, J68.4, J70.1, J70.3, J84.x, J92.x, J96.1, J98.2, J98.3, Z99.0, Z99.1 |  |
| Renal disease | E11.2, E13.2, E14.2, I12.x, I13.1, N00.x-N08.x, N17.x-N19.x, N25.x-N27.x | KASxx |
| Liver disease | B18.x, K70.x-K77.x | JJCxx |
| Venous thromboembolism (>1 year prior) | I26.x, I63.6, I67.6, I80.x-I82.x, I87.0, O22.3, O22.5, O87.1, O87.3, O88.2, Z86.7A, Z86.7B |  |
| Malignancy (>1 year prior) | C00.x-C97.x (not C44.x) |  |
| Peptic ulcer disease (>90 days prior) | K25.x-K28.x |  |
| Psychiatric disorder/substance abuse | E24.4, F06.x-F99.x (not F17.2), G31.2, G62.1, G72.1, I42.6, K29.2, K70.x, K86.0, O35.4, P04.3, Z71.4, Z86.4 |  |
| **Prescription-drug use (6-month look-back)^d^** | ***ATC code*** |  |
| ***Platelet inhibitors*** |  |  |
| Aspirin | B01AC06 |  |
| P2Y_12_ inhibitor (excl. prasugrel and ticagrelor) | B01AC04, B01AC05 |  |
| ***Other drugs*** |  |  |
| ACE inhibitor/ARB | C09A, C09B, C09C, C09D |  |
| Calcium-channel blocker | C08C, C08D |  |
| Loop diuretic | C03C, C03EB |  |
| Other diuretic | C03A, C03B, C03D, C03EA |  |
| Beta-blocker | C07 |  |
| Statin | C10AA, C10BA, C10BX |  |
| Metformin | A10BA02 |  |
| Insulin | A10A |  |
| Other glucose-lowering drug | A10B (not A10BA) |  |
| Antidepressant/antipsychotic | N05A, N06A |  |
| Beta_2_-agonist inhalant | R03AC |  |
| Anticholinergic inhalant | R03BB, R03AL |  |
| Glucocorticoid inhalant | R03BA, R03AK |  |
| Oral glucocorticoid | H02AB |  |
| NSAID | M01A |  |
| Opioid | N02A |  |
| **Health care utilization^c^** |  |  |
| No. of specialist care outpatient visits in the last year (0, 1-3, >3) |  |  |
| No. of hospital admissions in the last year (0, 1, >1) |  |  |
| No. of prescription drugs in the last year (0-5, 6-10, 11-15, >15) |  |  |

ACE, angiotensin-converting enzyme; AF, atrial fibrillation; ARB, angiotensin-receptor blocker; ATC, Anatomical Therapeutic Chemical classification system; ICD-10-SE, International Classification of Diseases, 10^th^ Revision, Swedish Edition; KVÅ, Classification of Care Measures; NSAID, nonsteroidal antiinflammatory drug; TIA, transient ischemic attack

^a^ Data from Statistics Sweden.

^b^ Missing values (<0.01% for place of birth, and <1% for education) were imputed with the mode.

^c^ Data from National Patient Register.

^d^ Data from Prescribed Drug Register.

**Table S5. Geographic baseline patient characteristics^a^**

| **Characteristic** | **DOAC use (n=103 703)** | **Comparator groups** | |
| --- | --- | --- | --- |
|  |  | **No DOAC use, AF (n=36 875)** | **No DOAC use, major CVD (n=355 699)** |
| **Population density of municipality, n (%)** |  |  |  |
| Large town | 22 283 (21.5) | 8087 (21.9) | 79 760 (22.4) |
| Commuter municipality close to major city | 17 620 (17.0) | 6353 (17.2) | 55 705 (15.7) |
| Commuter municipality close to large town | 17 684 (17.1) | 5870 (15.9) | 60 696 (17.1) |
| Major city | 13 954 (13.5) | 5582 (15.1) | 48 337 (13.6) |
| Small town | 15 710 (15.1) | 5428 (14.7) | 53 512 (15.0) |
| Commuter municipality close to small town | 7593 (7.3) | 2535 (6.9) | 26 363 (7.4) |
| Rural municipality | 6638 (6.4) | 2376 (6.4) | 24 479 (6.9) |
| Rural municipality with tourism sector | 2221 (2.1) | 644 (1.7) | 6847 (1.9) |
| **Health care region of residence, n (%)** |  |  |  |
| Stockholm | 18 974 (18.3) | 7023 (19.0) | 61 317 (17.2) |
| Västra Götaland | 17 023 (16.4) | 6564 (17.8) | 56 175 (15.8) |
| Skåne | 13 635 (13.1) | 5027 (13.6) | 49 914 (14.0) |
| Östergötland | 4396 (4.2) | 1572 (4.3) | 17 360 (4.9) |
| Jönköping County | 3399 (3.3) | 1171 (3.2) | 13 154 (3.7) |
| Gävleborg | 3211 (3.1) | 1325 (3.6) | 12 346 (3.5) |
| Uppsala | 3730 (3.6) | 1416 (3.8) | 11 308 (3.2) |
| Dalarna | 4315 (4.2) | 1128 (3.1) | 12 642 (3.6) |
| Halland | 4660 (4.5) | 1351 (3.7) | 11 884 (3.3) |
| Sörmland | 3274 (3.2) | 1126 (3.1) | 11 053 (3.1) |
| Värmland | 3826 (3.7) | 1100 (3.0) | 12 178 (3.4) |
| Norrbotten | 2860 (2.8) | 1067 (2.9) | 12 395 (3.5) |
| Västmanland | 3421 (3.3) | 839 (2.3) | 10 590 (3.0) |
| Örebro County | 3270 (3.2) | 1155 (3.1) | 10 873 (3.1) |
| Kalmar County | 2709 (2.6) | 990 (2.7) | 10 724 (3.0) |
| Västerbotten | 2569 (2.5) | 1156 (3.1) | 11 221 (3.2) |
| Västernorrland | 2501 (2.4) | 1060 (2.9) | 10 444 (2.9) |
| Blekinge | 1779 (1.7) | 546 (1.5) | 6526 (1.8) |
| Kronoberg | 2012 (1.9) | 569 (1.5) | 6162 (1.7) |
| Jämtland Härjedalen | 1316 (1.3) | 464 (1.3) | 5109 (1.4) |
| Gotland | 823 (0.8) | 226 (0.6) | 2324 (0.7) |

AF, atrial fibrillation; CVD, cardiovascular disease; DOAC, direct oral anticoagulant

^a^ Percentages may not total 100 because of rounding.

**Table S6. Risk of severe COVID-19 among DOAC users vs. non-use comparators according to DOAC subtype^a^**

| **Outcome** | **Events, n (%)** | **Unadjusted hazard ratio (95% CI)** | **Fully adjusted^b^ hazard ratio (95% CI)** |
| --- | --- | --- | --- |
| **Hospital admission for COVID-19** |  |  |  |
| Direct thrombin inhibitor use | 26 (0.25) | — | — |
| vs. non-use AF comparator | 95 (0.26) | 0.97 (0.63-1.50) | 0.74 (0.43-1.26) |
| vs. non-use CVD comparator | 1119 (0.31) | 0.80 (0.54-1.18) | 0.78 (0.52-1.17) |
| Direct factor Xa inhibitor use | 334 (0.36) | — | — |
| vs. non-use AF comparator | 95 (0.26) | 1.39 (1.11-1.74) | 1.03 (0.77-1.38) |
| vs. non-use CVD comparator | 1119 (0.31) | 1.14 (1.01-1.29) | 0.96 (0.82-1.13) |
| **ICU admission or death due to COVID-19** |  |  |  |
| Direct thrombin inhibitor use | 12 (0.12) | — | — |
| vs. non-use AF comparator | 55 (0.15) | 0.78 (0.42-1.45) | 0.58 (0.28-1.20) |
| vs. non-use CVD comparator | 473 (0.13) | 0.87 (0.49-1.55) | 0.81 (0.44-1.47) |
| Direct factor Xa inhibitor use | 149 (0.16) | — | — |
| vs. non-use AF comparator | 55 (0.15) | 1.07 (0.79-1.46) | 0.77 (0.52-1.15) |
| vs. non-use CVD comparator | 473 (0.13) | 1.20 (1.00-1.44) | 0.91 (0.71-1.16) |

AF, atrial fibrillation; CI, confidence interval; COVID-19, coronavirus disease 2019; CVD, cardiovascular disease; DOAC, direct oral anticoagulant; ICU, intensive care unit

^a^ The number of patients was 10 349 in the direct thrombin inhibitor use group, 93 354 in the direct factor Xa inhibitor use group, 36 875 in the non-use atrial fibrillation comparator group, and 355 699 in the non-use cardiovascular disease comparator group.

^b^ Adjusted for 42 potential confounders, including age, sex, sociodemographic factors, comorbidities, medications, and health care utilization (Table S4).

**Table S7. Risk of ICU admission and death due to COVID-19 analyzed separately^a^**

| **Outcome** | **Events, n (%)** | **Unadjusted hazard ratio (95% CI)** | **Fully adjusted^b^ hazard ratio (95% CI)** |
| --- | --- | --- | --- |
| **ICU admission due to COVID-19** |  |  |  |
| DOAC use | 40 (0.04) | — | — |
| vs. non-use AF comparator | 14 (0.04) | 1.02 (0.55-1.87) | 1.06 (0.48-2.35) |
| vs. non-use CVD comparator | 172 (0.05) | 0.80 (0.57-1.13) | 0.86 (0.55-1.34) |
| **Death due to COVID-19** |  |  |  |
| DOAC use | 140 (0.14) | — | — |
| vs. non-use AF comparator | 46 (0.12) | 1.08 (0.78-1.51) | 0.72 (0.47-1.10) |
| vs. non-use CVD comparator | 371 (0.10) | 1.30 (1.07-1.57) | 0.91 (0.70-1.17) |

AF, atrial fibrillation; CI, confidence interval; COVID-19, coronavirus disease 2019; CVD, cardiovascular disease; DOAC, direct oral anticoagulant; ICU, intensive care unit

^a^ The number of patients was 103 703 in the DOAC use group, 36 875 in the non-use atrial fibrillation comparator group, and 355 699 in the non-use cardiovascular disease comparator group.

^b^ Adjusted for 42 potential confounders, including age, sex, sociodemographic factors, comorbidities, medications, and health care utilization (Table S4).

**Table S8. All-cause mortality among DOAC users vs. non-use comparator groups**

|  | **Patients, n** | **All-cause deaths, n (%)** | **Unadjusted hazard ratio (95% CI)** | **Fully adjusted^a^ hazard ratio (95% CI)** |
| --- | --- | --- | --- | --- |
| DOAC use | 103 703 | 911 (0.88) | — | — |
| vs. non-use AF comparator | 36 875 | 311 (0.84) | 1.04 (0.92-1.18) | 0.62 (0.53-0.73) |
| vs. non-use CVD comparator | 355 699 | 2334 (0.66) | 1.34 (1.24-1.45) | 0.79 (0.71-0.87) |

AF, atrial fibrillation; CI, confidence interval; CVD, cardiovascular disease; DOAC, direct oral anticoagulant

^a^ Adjusted for 42 potential confounders, including age, sex, sociodemographic factors, comorbidities, medications, and health care utilization (Table S4).
